# Section of United Fracture and Wiring of the Ends

**Published:** 1876-08

**Authors:** 


Section of United Fracture and Wiring of the Ends.—Mr. A. Smith received a fracture of the right collar-bone from being thrown from a mule. The fragments had united with a lapping of one inch, and with more than the usual amount of deformity: The head of the humerus so pressed upon the nerves and blood-vessels of the axilla as to seriously interfere with the use of the right arm. Prof. Eve cut down upon the point of fracture by making a semilunar incision three inches in length, the convexity down, the middle of the incision reaching below the clavicle at the point of fracture. The flap was dissected up, exposing the overlapping fragments fully, and the subclavian artery and internal jugular vein. The viciouslyunited fragments were divided with a pair of cutting pliers, and the roughened ends squared off. The periosteum was cut through and a hole drilled in each fragment half an inch from the end, a silver wire passed through, and the ends of the fragments brought into apposition by pressing the shoulder backward, outward, and upward. The wire was then tightly tied and the ends thrust into the holes. The wound was closed with sutures and adhesive plaster, and dressed with carbolic lotion. A soft pad was placed in the axilla, and the hand carried across the chest and placed in a sling. The patient suffered from ague and erysipelas after the operation, but got well, with a fine result. To use Prof. Eve's words: "And why not? for who does not know the innocuity of silver wire to the flesh, and how easily it may become incased in callus? I, therefore, recommend the metallic suture for all fractures of the clavicle, recent or otherwise, believing that the slight exposure made by the operation as described would not much increase the danger in such cases, wherein the methods now resorted to fail to keep the ends of the broken bones in apposition."—*New York Medical Journal.*

